# Experience from creating electronic systems with generic endpoint review and adjudication flows

**DOI:** 10.1186/1745-6215-16-S2-O61

**Published:** 2015-11-16

**Authors:** Jane Aziz, Sharon Kean, Ernest Edifor, Eleanor Dinnett

**Affiliations:** 1 University of Glasgow, Glasgow, UK

## Background

An increasing number of Clinical Trial Web Portals now include systems to manage endpoint adjudication. These cover the entire process from the initial identification of potential endpoints, the creation of electronic endpoint packages, and assignment of endpoints to members of the relevant Clinical Endpoint Committee (CEC) to the final adjudication verdict being entered into the system. The number and types of users involved at each stage and the number and size of Endpoint Committees varies across trials, depending on the trial design and requirements of the Sponsor, so the challenge has been to create generic systems that can handle the variations in design.

## Methods

By separating the roles that the system performs from the roles a user may have within a trial the differences in the users that are involved can be dealt with and a generic flow created. Handling differences in the adjudication process, such as how many committee members are initially assigned, what happens if they are not in agreement etc., are more complex but can be mostly overcome. Additional complexity is introduced where the actual processes involved are different or not required at all for a particular trial.

## Conclusion

We will discuss the different models and technological solutions applied to overcome complex trial specific processes in order to achieve a generic system. Serious gain can now be made with the provision of generic systems that reduce programming effort and training. The use of electronic adjudication also saves a considerable amount of time in determining trial outcomes.

